# Music-evoked EEG signals for mental health assessment using two- and three-class classification

**DOI:** 10.3389/fnsys.2026.1886805

**Published:** 2026-09-23

**Authors:** Abdelkader Nasreddine Belkacem, Abdelhadi Hireche, Nour Faris Ali, Mohamed Atef, Abdalla A. R. M. Hamid, Ibrahim M. Elfadel, Herbert F. Jelinek

**Affiliations:** 1 Department of Computer and Network Engineering, United Arab Emirates University, Al Ain/Abu Dhabi, United Arab Emirates; 2 Biomedical Engineering Program, College of Engineering, American University of Sharjah, Sharjah, United Arab Emirates; 3 Electrical and Communication Engineering Department, College of Engineering, United Arab Emirates University, Al Ain/Abu Dhabi, United Arab Emirates; 4 Department of Clinical Psychology, United Arab Emirates University, Al Ain/Abu Dhabi, United Arab Emirates; 5 Department of Computer and Information Engineering, Khalifa University, Abu Dhabi, United Arab Emirates; 6 Department of Medical Sciences, Khalifa University, Abu Dhabi, United Arab Emirates

**Keywords:** anxiety, deep learning, deep residual MLP, depression, electroencephalography (EEG), mental fatigue, music therapy assessment, stress

## Abstract

**Introduction:**

Electroencephalography (EEG), a non-invasive method of measuring brain activity, has become a valuable tool in mental health research for examining neural activity associated with various psychological disorders and neurofeedback therapies, including music therapy. However, the use of EEG under music and no-music conditions for mental health screening remains insufficiently validated.

**Methods:**

In this preliminary study, we implemented an experimental protocol in which 48 participants underwent EEG recordings during both music and no-music sessions with identical relaxation instructions. The Patient Health Questionnaire-9 (PHQ-9) and the Generalized Anxiety Disorder 7-item (GAD-7) scale were also included. Five complementary EEG features were extracted for each mental fatigue class: power spectral density (PSD) across different frequency bands, band powers, alpha-theta ratio, theta-beta ratio, and channel coherence. Binary classification (mental fatigue) and multi-output binary classification (anxiety, depression, and stress components) were implemented using a deep residual multi-layer perceptron (MLP), a feedforward neural network with residual skip connections. Leave-Subject-Out cross-validation ensured robust generalization, with all metrics computed at the subject level rather than epoch level.

**Results:**

For binary classification, the model achieved 72.92% accuracy in the music condition and 58.33% accuracy in the no-music condition, with notably high recall (96.67%) in the music condition. Multi-output classification in the music condition showed stress with high accuracy (93.75%) but low AUC (35.56%), reflecting the high prevalence of the stressed class (85.7%), while anxiety and depression presented greater challenges (54.17% and 62.50% accuracy, respectively).

**Discussion:**

These preliminary results suggest that music may modulate brain activity patterns in ways that enhance the discriminability of mental fatigue states, although the fixed condition order means order and habituation effects cannot be ruled out.

## 1 Introduction

Mental health disorders, including conditions such as anxiety and depression, as well as related states such as persistent stress, constitute a major global health challenge, affecting millions of people and their quality of life, productivity, and general wellbeing (World Health Organization, 2003). Conventional mental health evaluation methods are based primarily on self-reported questionnaires and clinical interviews. Commonly used tools include the seven-item generalized anxiety disorder scale (GAD-7) (Spitzer et al., 2006), the patient health questionnaire-9 (PHQ-9) (Kroenke et al., 2001), the multidimensional fatigue scale (MFS) (Smets et al., 1995) and the perceived stress scale (PSS) (Cohen et al., 1983), all of which help quantify mental health status. Although these instruments are effective for screening and preliminary diagnosis, they are intrinsically subjective and susceptible to numerous biases, such as social desirability, recall bias, and the present emotional state of the participant (Kohrt et al., 2016; Kroenke et al., 2021). In this study, we use “mental fatigue” as the primary outcome, operationalized as an umbrella construct encompassing self-reported symptoms of anxiety, depression, and stress as measured by these standardized questionnaires.

Electroencephalography (EEG) is a non-invasive measurement method for recording electrical brain activity and has become a valuable tool in mental health research to examine neural activity associated with various psychological disorders (Hammond, 2005; Lei et al., 2022; Masri et al., 2023; Belkacem et al., 2020, 2023; Jamil et al., 2021). Distinct EEG wave patterns, categorized into gamma, alpha, beta, theta, and delta frequency bands, have been linked to specific psychological states and cognitive processes. For instance, frontal alpha asymmetry has been frequently associated with depression, indicating increased relative right hemispheric activity (Sun et al., 2017). Similarly, elevated beta activity has been associated with anxiety states, with increased beta wave activity (13–30 Hz), especially high beta (>20 Hz), found in anxiety conditions (Leaf et al., 2023). EEG patterns have also been associated with mental fatigue showing an increase in theta frequency band and reduced alpha frequency band power (Othmani et al., 2023). By analyzing such neural patterns, EEG can provide objective information on mental health, complementing traditional survey-based assessments (Belkacem et al., 2020, 2023).

Music significantly influences emotional states and brain activity. Changes in EEG patterns can reflect mood, arousal, and cognitive processing (Cui et al., 2022). Furthermore, EEG analyzes have shown that listening to music can alter brain activity, suggesting a stimulating effect on listeners (Su et al., 2024). Asif et al. (2019) conducted a study that examined the effects of music tracks in English and Urdu on human stress levels using EEG signals and developed a classification system for the identification of stress. Recent studies have also explored how music affects resistance to mental fatigue using EEG power spectrum analysis, indicating potential benefits in mitigating fatigue-related cognitive decline (Liu et al., 2025).

Music therapy (MT) has been shown to reduce depression and anxiety symptoms in depression and comorbid anxiety correlated with increased power in the alpha and theta frequency bands, indicating neural reorganization associated with reduced depression and anxiety (Fachner et al., 2013).

Despite this therapeutic evidence, music has rarely been exploited as a recording paradigm for mental health screening. Most EEG-based mental health studies rely on conventional resting-state recordings, in which neural activity reflects a relatively stable, unstimulated baseline. Music listening, by contrast, engages attention, emotion, and memory networks and induces broader, condition-specific neural dynamics (Koelsch, 2014; Menon and Levitin, 2005). We hypothesize that these music-evoked responses amplify fatigue-related differences in brain activity and therefore expose more discriminative biomarkers than resting-state EEG alone. This study accordingly evaluates music not as a therapeutic intervention but as a recording context that may enhance the separability of mental fatigue states.

Machine learning approaches have been increasingly used to elucidate the relationships among EEG-derived neural signatures, cognitive and motor function (Chen et al., 2021a; Belkacem and Lakas, 2021; Shuqfa et al., 2023; Liang et al., 2022), and psychopathological phenotypes (Chen et al., 2023; Ali et al., 2024). A computer-aided model has been proposed for automatic classification of mental health status using the alpha band of EEG signals, while another approach has focused on developing an efficient model for depression detection using EEG, incorporating an encoder, long short-term memory, and an attention mechanism, achieving high accuracy on epileptic seizure and schizophrenia datasets (Ranjan et al., 2022; Ali et al., 2024). The integration of AI techniques, including deep learning and multimodal fusion, has shown promise in improving mental health assessments (Rajpurkar et al., 2022). Additionally, wearable AI-based approaches have been explored to monitor anxiety and depression, integrating EEG and other physiological signals for improved mental health assessments (Abd-Alrazaq et al., 2023; Chen et al., 2021c,b). Dutta et al. (2020) utilized reinforcement learning with EEG signals to personalize music therapy for emotion management, demonstrating the potential of adaptive systems in therapeutic interventions. Several studies have explored the use of AI and EEG in assessing the effect of music on depression and anxiety; however, research on mental fatigue and the application of AI to evaluate the effect of music on mental fatigue remains sparse (Ding, 2022; Karim et al., 2023).

Several studies have investigated the application of EEG-based approaches in music therapy and mental health assessment, as summarized in Table 1. These works demonstrate the potential of EEG features for classifying various mental states and emotions in response to music stimulation, with classification accuracies ranging from 64.28% to 99.31% depending on the complexity of the task and features used.

**Table 1:** Summary of related works on EEG-based music therapy and music-related physiological studies.

References	Purpose	Methods	EEG features	Classifier	Results
Lee et al. (2020)	Develop music recommendation system for depression therapy using EEG	EEG via Bluetooth headset (alpha, beta waves), deep learning (CNN, NAS), music verification	Alpha and beta wave changes	CNN, NAS	CNN: 99.31% accuracy in depression classification; robust recommendations
Rahman et al. (2021)	Explore music genre effects on physiological signals for mental health	EDA, BVP, ST, and PD from 24 participants, 12 music pieces (three genres), 34 features, NN classification	N/A (physiological signals only)	Neural Network	99.2% genre classification, 98.5% emotion accuracy; EDA most effective
Zhou et al. (2022)	Analyze music effects on emotions using EEG for psychological healthcare	32-channel EEG, EER model (20 emotions), MEC model (eight emotions), and semantic mapping	Power spectral density (delta, theta, alpha, beta, and gamma bands)	CNN	EER: >90% (binary), >80% (multi-class); MEC: >90%; joy/sadness differentiation
Arsalan et al. (2019)	Classify perceived mental stress using commercial EEG headband	28 participants, four-channel MUSE headband, pre/post phases, PSS, five feature groups	PSD, correlation, asymmetry, and power spectrum (five frequency bands)	SVM, Naive Bayes, and MLP	92.85% (binary), 64.28% (ternary) accuracy; pre-activity phase optimal
Asif et al. (2019)	Classify stress using EEG responses to music; assess genre/language effects	27 participants, four-channel MUSE, STAI assessment, five feature groups, four classifiers	Absolute power, relative power, coherence, phase lag, asymmetry (five bands)	SMO, SGD, LR, and MLP	LR: 98.76% (binary), 95.06% (ternary); English music more effective
Li et al. (2025)	Analyze effects of music genres on driving stress using EEG	60 drivers, 120 EEG recordings, simulated driving, four music genres	PSD, time-frequency features	SVM, KNN, and RF	90% accuracy in stress classification; music genre effects evaluated
Liu and Zhao (2025)	EEG Mind-Transformer for mental health monitoring	DT-GAM, HGRA, and STFM for EEG spatiotemporal analysis	Raw EEG features (transformer)	Transformer (DT-GAM)	State-of-the-art on mental health EEG benchmarks

Building on the rationale above, our paper evaluates whether music-evoked EEG provides more discriminative biomarkers of mental fatigue than recordings without music, and whether specific EEG traits are linked to music-related changes in brain activity. We present a comprehensive pipeline for mental health classification using EEG features and a deep residual multi-layer perceptron (MLP), a feedforward neural network architecture with multiple hidden layers. Our approach includes two classification frameworks: (1) binary classification (two-class) for the primary mental fatigue outcome, and (2) multi-output binary classification for simultaneous prediction of the anxiety, depression, and stress components that underpin this outcome, where each of the three output heads produces an independent binary prediction for its respective component. We employ Leave-Subject-Out Cross-Validation (LOSO-CV) to ensure robust generalization to unseen individuals, with all metrics computed at the subject level rather than epoch level. This work represents a step toward objective, AI-driven EEG-based mental health screening that could enable early detection and more effective monitoring of mental health conditions. However, these findings are preliminary and are not intended for clinical diagnosis.

## 2 Materials and methods

### 2.1 Experimental paradigm and protocol

This study involved human participants. Approval of all ethical and experimental procedures and protocols was granted by UAE University Human Research Ethics Committee (UAEU-HREC) under Application No. ERH_2022_2148 2022_20.

Forty-nine subjects were recruited for this experiment from the United Arab Emirates University. One subject was excluded due to incomplete questionnaire data, yielding *n* = 48 for analysis (30 females, 18 males; age range 18–25 years). Subjects were briefed about the objectives and procedures of the study before providing their informed consent. Each participant completed a set of standard self-report questionnaires (GAD-7, MFS, PHQ-9, and PSS) for 15 mins. The personal information of the subjects was anonymized and saved in a password-protected storage space. Upon examining the responses of each participant to the standard self-report questionnaires, a licensed clinical psychologist (author A.A.R.M.H.), blinded to the EEG data, classified each participant into the mental-fatigue or non-mental-fatigue group using a standardized fatigue assessment. Group assignment was based on the score of a validated fatigue questionnaire (the Multidimensional Fatigue Scale, MFS) and a predefined cutoff value, rather than on subjective clinical judgment alone. Each subject participated in a 5-min EEG recording session without auditory stimulation, followed by another 5-min recording with auditory stimulation, as illustrated in Figure 1. The order of conditions was fixed (no-music followed by music) for all participants. The auditory stimulus used during the music condition was the track *Ambiance* by REIKI WORLD (Ambiance—Single, 2021; duration: 9:07; genre: Alternative/Ambient), available at https://music.apple.com/ng/album/ambiance-single/1552802573. The music was played through a Jabra Speak 510 speaker positioned in front of the participant. A g.USB amplifier (g.tec Medical Engineering, Schiedlberg, Austria) with a 16-channel dry electrode cap was used to record EEG data according to the 10–20 system. The electrode positions included Fp1, Fp2, F7, F3, Fz, F4, F8, T7, Cz, T8, P7, P3, P4, P8, O1, and O2. EEG data was sampled at 1,200 Hz. Subjects were requested to remain calm and still during the EEG sessions to reduce movement artifact. Participants were seated in a quiet, closed room with low ambient noise. Immediately before the start of each recording session, participants were instructed to keep their eyes closed for the entire recording. Eye-opening episodes were detected retrospectively from the EOG-correlated components and removed during the ICA-based artifact rejection described below. A 1-min break separated the two conditions. Movement artifacts were not monitored in real time.

**Figure 1:**
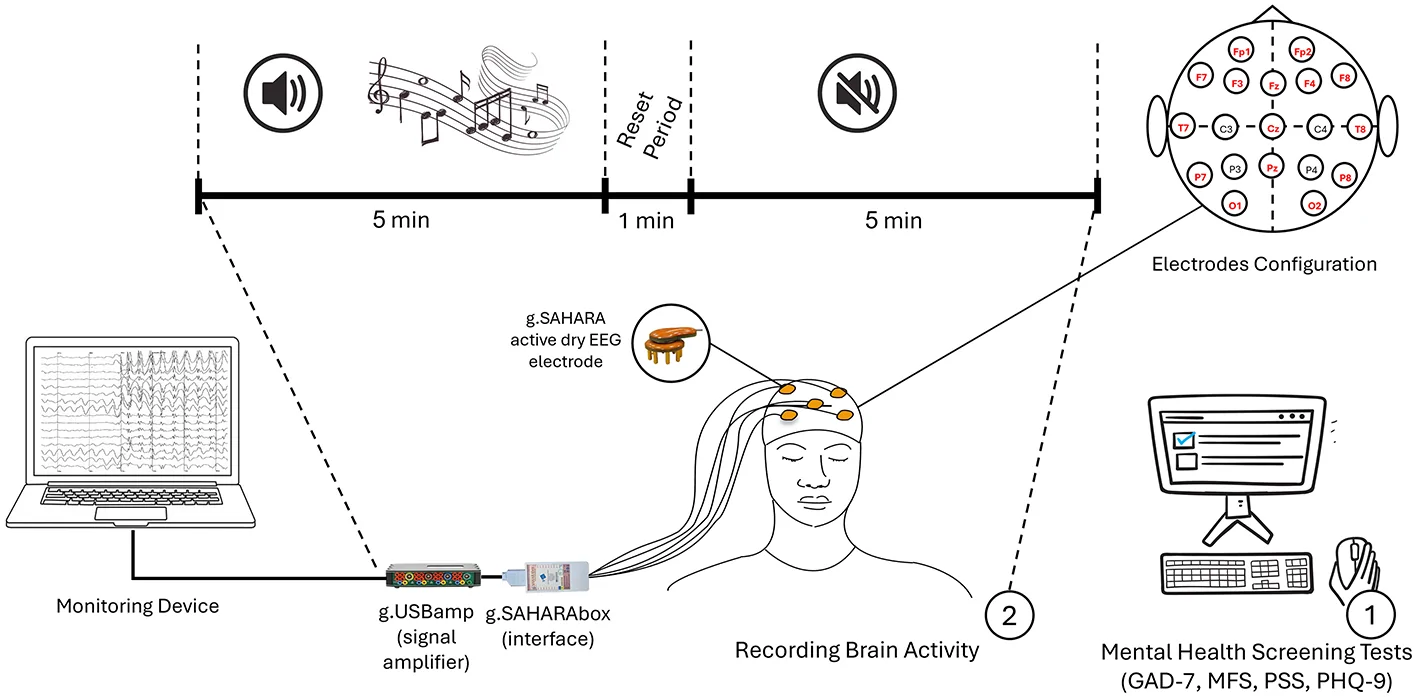
Overview of the experimental protocol and setup. The timeline shows two 5-mins EEG recording sessions (without and with music) separated by a 1-min reset period. Components include: (1) Mental health screening using standardized questionnaires, (2) EEG recording setup with electrode configuration following the 10–20 system, and real-time monitoring equipment.

### 2.2 Preprocessing

To improve data quality, EEG signals were filtered within the 1–45 Hz range using a fourth-order Butterworth bandpass filter with zero-phase implementation (filtfilt) to remove high-frequency noise and low-frequency drift. Furthermore, power line frequency noise was removed using a 50 Hz notch filter (Q-factor: 30). Artifact removal was performed using independent component analysis (ICA) using the fastICA library (Hyvärinen and Oja, 2000), which decomposed the signal into 15 components; artifact-related components were automatically identified using EOG correlation, muscle artifact detection, and kurtosis-based outlier detection, with a maximum of six components excluded. The complete preprocessing pipeline is illustrated in Supplementary Figure 1 for a representative subject. Furthermore, each 5-min EEG recording was segmented into 10-s epochs with no overlap, and each epoch was individually analyzed.

### 2.3 Frequency-domain analysis

To examine changes in EEG patterns associated with mental fatigue, we initially performed a group frequency analysis. The initial step involved estimating the power spectral density (PSD) across all major EEG frequency bands (delta, theta, alpha, beta, and gamma) for both groups (with and without mental fatigue) under each condition (with and without music). PSD averages at the group level were calculated for each frequency band, allowing an extensive analysis of power distribution variations in relation to mental fatigue and auditory conditions.

### 2.4 Feature extraction

Five complementary features were extracted to analyze EEG data on mental fatigue, each capturing different aspects of brain activity. All features were calculated for each segmented 10-s epoch across all EEG channels:

**Power Spectral Density (PSD):** PSD measures the allocation of power among EEG frequency bands. For every 10-s epoch, the power spectral density was calculated using the Welch method with a 2-s window and 50% overlap. PSD values were computed separately for each EEG channel, concentrating on the 4–30 Hz frequency range (encompassing theta, alpha, and beta bands) to capture fine-grained spectral shape. Band-power features (see below) were separately computed for all five canonical bands as integrated power values, providing complementary summary statistics per band.

**Band powers:** Absolute power was computed for each standard EEG frequency band (delta: 0.5–4 Hz, theta: 4–8 Hz, alpha: 8–13 Hz, beta: 13–30 Hz, and gamma: 30–100 Hz) by integrating the PSD over the respective frequency ranges for each channel. Gamma band (30–100 Hz) features were included but interpreted with caution, as dry electrodes are susceptible to electromyographic (EMG) artifacts at these frequencies.

**Alpha-theta ratio:** The ratio of alpha band power to theta band power was calculated for each channel, as this metric has been associated with cognitive states and mental fatigue.

**Theta-beta ratio:** Similarly, the ratio of theta to beta band power was computed for each channel, providing additional information about arousal and attention states.

**Coherence:** Functional connectivity was evaluated by calculating the coherence between all pairs of EEG channels within the 4–30 Hz frequency range for each epoch. Coherence measures the phase consistency between signals, reflecting the degree of synchronization between brain regions. Coherence was calculated and averaged for each channel pair within the designated frequency band, yielding a symmetric 16 × 16 coherence matrix for each epoch.

These five feature types were concatenated to form a comprehensive feature vector for each epoch, capturing both spectral characteristics and functional connectivity patterns.

### 2.5 Model architecture: deep residual MLP

We employed a deep residual multi-layer perceptron (MLP), a feedforward neural network architecture that incorporates residual (skip) connections to improve gradient flow and enable training of deeper networks. The deep residual MLP progressively transforms the input feature vector through successive layers, with skip connections allowing the network to learn both shallow and deep feature representations that capture complex, non-linear relationships between EEG features and mental health outcomes. Residual MLPs are well-suited for tabular data and provide a strong baseline for classification tasks, offering a balance between model capacity and computational efficiency.

#### 2.5.1 Deep residual MLP architecture

The deep residual MLP processes the concatenated feature vector (comprising PSD, band powers, alpha-theta ratio, theta-beta ratio, and coherence features) through a series of fully-connected hidden layers with residual skip connections. The architecture consists of:

**Input layer:** Receives the comprehensive EEG feature vector combining all five feature types (PSD, band powers, alpha-theta ratio, theta-beta ratio, and coherence) across all 16 EEG channels. Input features are first normalized via batch normalization, then projected to the first hidden dimension through a fully-connected layer.

**Residual blocks:** The core of the architecture consists of three residual blocks operating at the first hidden dimension (512 neurons). Each residual block contains two fully-connected layers with batch normalization and ReLU activation, with a skip connection that adds the block's input to its output. This design mitigates the vanishing gradient problem and allows the network to learn identity-like mappings when beneficial, improving training stability for deep architectures.

**Hidden layers:** The network comprises three hidden dimensions: 512, 256, and 128 neurons. After the input projection to 512 dimensions, three residual blocks process features at this dimension. Two downsampling layers then progressively reduce the dimensionality (512 → 256 and 256 → 128), each consisting of a linear layer followed by batch normalization, ReLU activation, and dropout.

**Dropout regularization:** Dropout (0.3) is applied after each residual block and downsampling layer during training to prevent overfitting by randomly deactivating a proportion of neurons, forcing the network to learn redundant and robust feature representations.

**Output layer:** A single neuron with sigmoid activation produces the final binary prediction probability for mental fatigue classification. For multi-output classification, the final hidden representations are fed into separate output heads for each mental health component.

This architecture provides a strong baseline for EEG-based classification while maintaining interpretability through *post-hoc* feature importance analysis methods such as SHAP (SHapley Additive exPlanations).

### 2.6 Training configuration

The deep residual MLP model was trained using binary cross-entropy loss and the Adam optimizer (Diederik, 2014). Hyperparameters were selected through preliminary experiments and varied across different experimental conditions:

**Learning rate:** Ranged from 10^−5^ to 10^−3^ depending on the specific experimental configuration, with lower learning rates used for more stable convergence in challenging cases.

**Batch size:** Varied from 8 to 256 samples, with smaller batch sizes generally used when computational memory was limited or when working with imbalanced data after SMOTE augmentation.

**Training epochs:** Models were trained for 50–70 epochs, with early stopping implemented to prevent overfitting. Early stopping monitored validation loss with a patience of five epochs and a minimum improvement threshold of 0.001, automatically terminating training when no improvement was observed.

**Regularization:** Dropout (0.3) was applied after each hidden layer in the deep residual MLP architecture to prevent overfitting. Additionally, SMOTE was applied exclusively to each training fold within the LOSO-CV procedure to address class imbalance, ensuring balanced representation of both mental fatigue states during training. The held-out test subject's data was never seen during SMOTE fitting or any preprocessing step, preventing data leakage between training and evaluation.

### 2.7 Binary classification for mental fatigue detection

The primary classification task involves binary classification to distinguish between participants with and without mental fatigue. We employed a deep residual MLP, a feedforward neural network with multiple hidden layers and residual skip connections, which provides a robust baseline for EEG-based classification tasks.

#### 2.7.1 Leave-subject-out cross-validation

To ensure robust generalization and avoid subject-specific bias, we implemented Leave-Subject-Out Cross-Validation (LOSO-CV). In this approach, each of the 48 subjects serves as the test set exactly once, while all remaining 47 subjects form the training set. This strategy ensures that the model is evaluated on completely unseen subjects, providing a realistic estimate of performance on new individuals.

#### 2.7.2 Training configuration

The deep MLP model was trained using binary cross-entropy loss with the Adam optimizer. Given the class imbalance in the dataset, SMOTE (Synthetic Minority Over-sampling Technique) was applied to each training fold independently to balance the class distribution. Hyperparameters varied across experiments: batch size ranged from 8 to 256, learning rate from 10^−5^ to 10^−3^, and training epochs from 50 to 70. Early stopping was implemented with a patience of 5 epochs and minimum improvement threshold of 0.001 to prevent overfitting.

#### 2.7.3 Subject-level aggregation

Rather than evaluating performance at the epoch level, we aggregated predictions at the subject level to obtain more clinically meaningful metrics. For each test subject, predicted probabilities from all their epochs were averaged to produce a single probability score. The final binary prediction was determined by thresholding this averaged probability at 0.5. This aggregation strategy ensures that the reported metrics reflect subject-level classification performance rather than epoch-level performance, which is more relevant for clinical applications.

All performance metrics (accuracy, precision, recall, F1 score, and AUC) were computed based on these subject-level predictions across all 48 subjects, providing a robust evaluation of the model's ability to generalize to new individuals.

### 2.8 Multi-output classification for mental health components

Building upon the deep residual MLP architecture, we implemented a multi-output classification approach to simultaneously predict anxiety, depression, and stress. This extension allows us to analyze how individual mental health components are reflected in EEG patterns and provides insights into the relationships between these components.

For this analysis, we modified the deep residual MLP architecture to include three parallel output heads, each corresponding to a specific mental health component (anxiety, depression, and stress). Each output head consists of a dedicated fully-connected layer with sigmoid activation that processes the final hidden representations independently. The training process employed the same Leave-Subject-Out cross-validation strategy and followed similar hyperparameter configurations as the binary classification task.

The multi-output loss function was defined as a weighted sum of individual binary cross-entropy losses for each component, allowing the model to jointly optimize all three predictions while potentially sharing useful feature representations across tasks. This approach enabled us to predict individual mental health components and analyze their inter-relationships, providing a more comprehensive assessment framework.

The complete data processing and classification pipeline is presented in Algorithm 1.

Algorithm 1 EEG-based mental health classification pipeline. 
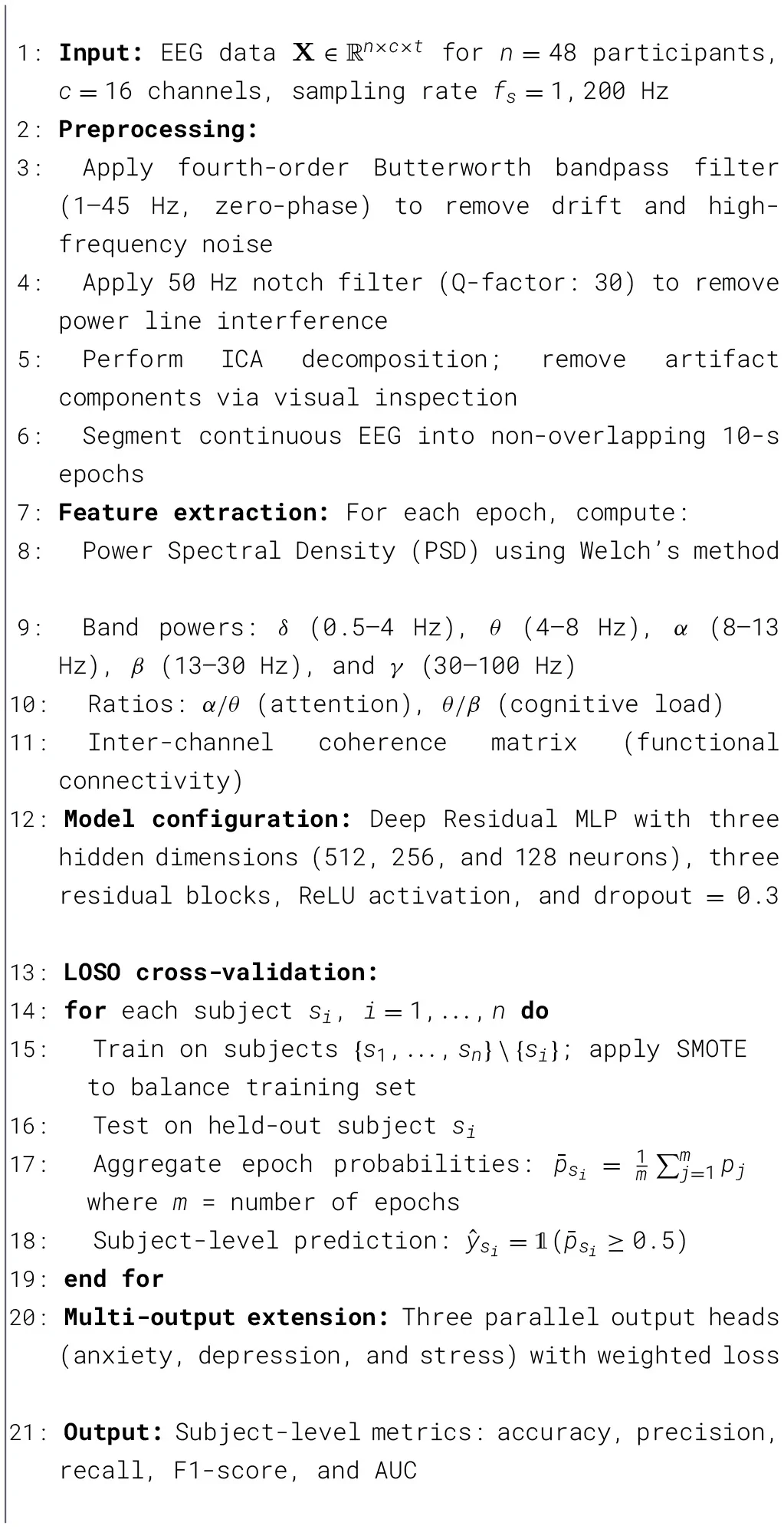

## 3 Results

This section presents the results of our EEG-based mental fatigue classification study using a deep residual MLP architecture with Leave-Subject-Out cross-validation. We first analyze the characteristics of extracted features under different conditions, followed by the performance evaluation of our subject-level classification approach for mental fatigue detection. Finally, we present the results of our multi-output classification extension for predicting individual mental health components. All analyses were conducted using the experimental data from 48 subjects (one subject excluded due to incomplete questionnaire data) under both music and no-music conditions, with all metrics computed at the subject level.

### 3.1 Feature analysis

#### 3.1.1 Power Spectral Density (PSD) of brain activation

Our analysis of EEG spectral patterns revealed distinct characteristics between the two groups with and without mental fatigue and with and without music stimulation. In the absence of music, subjects without mental fatigue demonstrated a higher overall power and notably fewer signal variability across frequency bands, as shown by the narrower confidence intervals in their power spectral plots in Figure 2 (first row plots). These subjects exhibited higher power in the alpha band. In contrast, those with mental fatigue exhibited increased theta activity in the frontal areas, along with greater signal variability, as indicated by wider confidence intervals in their spectral plots. Beta activity showed notable differences between the groups, with a higher frontal beta power observed in the fatigue group.

**Figure 2:**
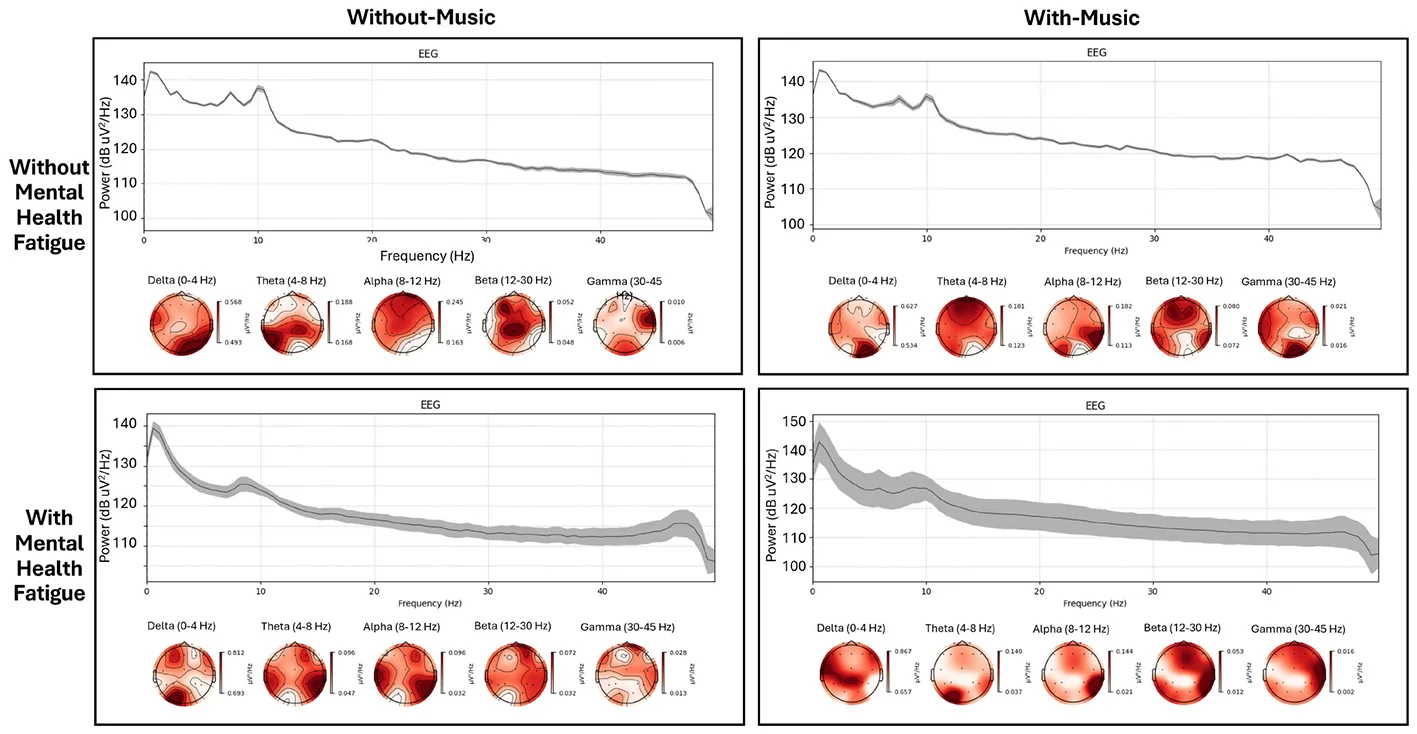
EEG spectral power distribution and topographic maps across frequency bands (delta, theta, alpha, beta, and gamma) for participants with and without mental fatigue, shown in music and no-music conditions. The line plots show power spectral density (PSD) with confidence intervals, while the topographic maps illustrate the spatial distribution of power for each frequency band. Darker red indicates higher power values.

The fatigue group showed reduced theta power in music condition compared to without-music condition, while the activity of the alpha band became more prominent in the posterior regions for both groups. In particular, subjects without mental fatigue maintained their characteristic pattern of higher power and lower variability even during music stimulation, suggesting a more stable baseline brain state. Interestingly, beta-band differences between groups were less pronounced with music stimulation, indicating a potential normalizing effect of music on cortical arousal. Individual PSD features are shown in Supplementary Figure 3 for selected representative subjects. Additional spectral power distributions and topographic maps for representative participants are provided in Supplementary Figure 2.

To quantify the statistical significance of these observed differences, we conducted independent-samples *t*-tests on the individual 10-s epochs pooled across subjects within each group (with and without mental fatigue), separately for each EEG channel and each condition. Table 2 presents the comprehensive statistical analysis results.

**Table 2:** Epoch-level *t*-test results for with and without music cases between group 1 (without mental fatigue) and group 2 (with mental fatigue).

Channel	Without music		With music	
	*t*-statistic	*p*-value	*t*-statistic	*p*-value
Fp1	12.754016	3.41 × 10^−37^ < 0.001	13.716292	9.62 × 10^−43^ < 0.001
Fp2	11.808970	3.89 × 10^−32^ < 0.001	12.589835	2.69 × 10^−36^ < 0.001
F7	12.753030	3.45 × 10^−37^ < 0.001	6.221693	4.93 × 10^−10^ < 0.001
F3	12.661342	1.11 × 10^−36^ < 0.001	11.477047	1.83 × 10^−30^ < 0.001
Fz	11.724832	1.05 × 10^−31^ < 0.001	13.648761	2.44 × 10^−42^ < 0.001
F4	11.593327	4.89 × 10^−31^ < 0.001	12.124892	8.47 × 10^−34^ < 0.001
F8	10.024158	1.25 × 10^−23^ < 0.001	9.250764	2.28 × 10^−20^ < 0.001
T7	5.400317	6.67 × 10^−08^ < 0.001	–3.099572	1.94 × 10^−03^
Cz	10.995009	4.32 × 10^−28^ < 0.001	12.112560	9.99 × 10^−34^ < 0.001
T8	11.534954	9.57 × 10^−31^ < 0.001	12.786297	2.20 × 10^−37^ < 0.001
P7	12.681912	8.55 × 10^−37^ < 0.001	11.122921	1.03 × 10^−28^ < 0.001
P3	9.205291	3.49 × 10^−20^ < 0.001	–1.636416	1.02 × 10^−01^
P4	11.270358	1.99 × 10^−29^ < 0.001	1.133915	2.57 × 10^−01^
P8	10.676664	1.40 × 10^−26^ < 0.001	12.797275	1.93 × 10^−37^ < 0.001
O1	10.476262	1.17 × 10^−25^ < 0.001	3.697266	2.18 × 10^−04^
O2	8.367592	6.04 × 10^−17^ < 0.001	4.474280	7.67 × 10^−06^ < 0.001

Tests were performed on individual 10-s epochs pooled across subjects.

The statistical analysis reveals highly significant differences between groups across most EEG channels in both conditions. Because these tests pool all epochs across subjects, epochs within the same subject are not statistically independent, and the resulting *p*-values are therefore anti-conservative; they should be interpreted as exploratory evidence that warrants confirmation with subject-level analyses (one observation per subject per channel per condition). To account for multiple comparisons across 16 channels and two conditions (32 tests), we applied Bonferroni correction (α = 0.05/32 = 0.00156). After correction, all 16 channels remained significant in the no-music condition, while three channels in the music condition lost significance: T7 (*p*_Bonf_ = 0.062), P3 (*p*_Bonf_>0.99), and P4 (*p*_Bonf_>0.99). Benjamini–Hochberg FDR correction yielded consistent results, with only P3 and P4 in the music condition failing to reach significance. Notably, the frontal channels (Fp1, Fp2, F3, Fz, and F4) consistently showed the strongest statistical differences, supporting our observations of prominent frontal activity changes in mental fatigue. The loss of significance at T7, P3, and P4 specifically in the music condition suggests that music may normalize neural activity patterns in temporal and parietal regions.

#### 3.1.2 Coherence features

Coherence analysis (Figure 3) revealed differences in functional connectivity patterns between groups. In the without-music condition, significant differences (marked in red in the difference matrices) were observed across multiple channel pairs, suggesting altered neural synchronization in mental fatigue. The coherence patterns were particularly distinct in the frontal-parietal connections. With music stimulation, while significant differences in coherence patterns persisted, they showed a different spatial distribution, with more pronounced differences in temporal and central channel pairs. These findings suggest that mental fatigue affects both local spectral power and large-scale functional connectivity patterns, with music modulating these differences in specific frequency bands and brain regions. Individual coherence features are shown in Supplementary Figure 4 for selected representative subjects.

**Figure 3:**
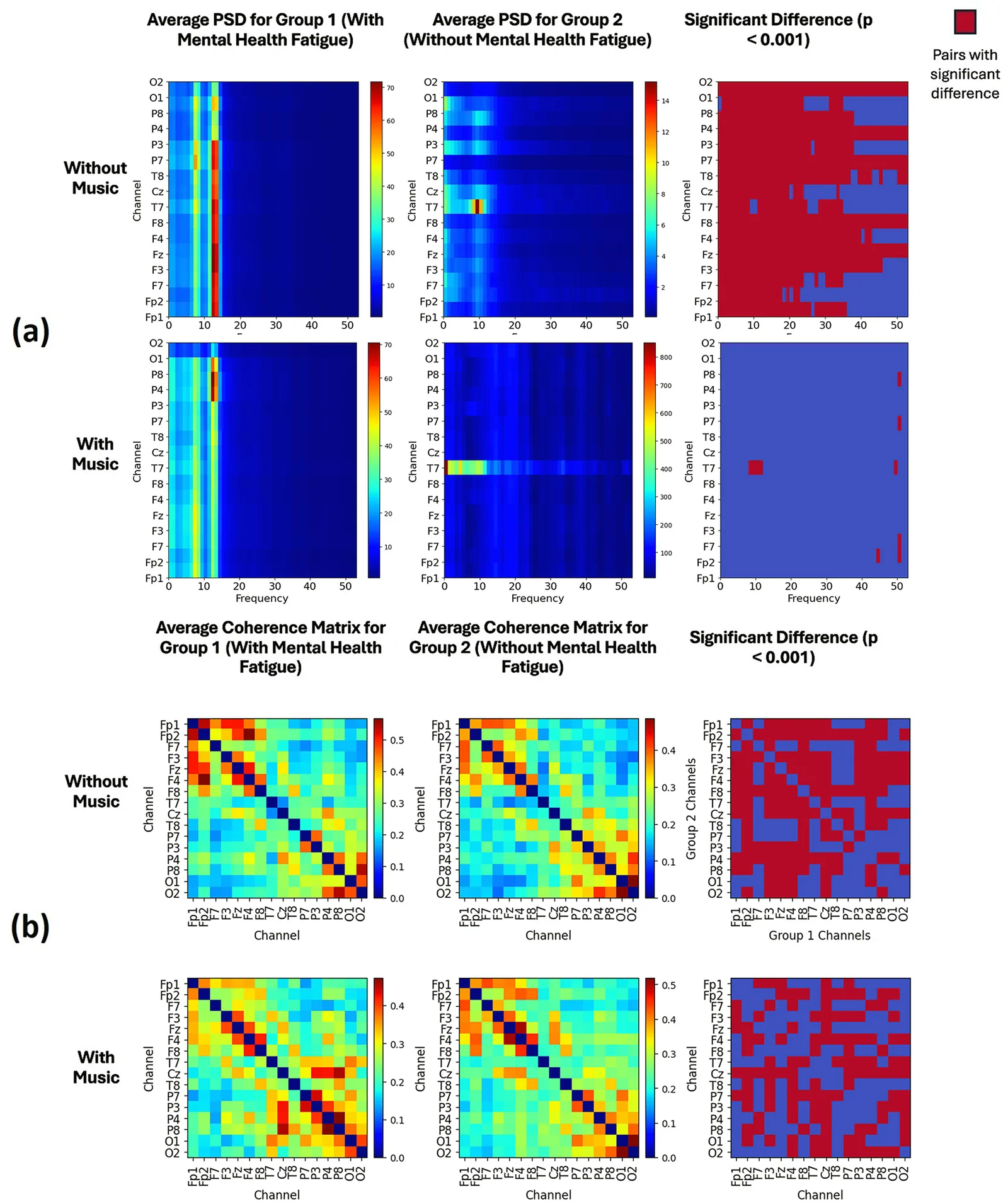
Group-level EEG spectral and functional-connectivity differences between participants with and without mental health fatigue, under without-music and with-music conditions. **(a)** Average power spectral density (PSD) heatmaps across EEG channels (y-axis) and frequency (x-axis) for Group 1 (with mental health fatigue) and Group 2 (without mental health fatigue), together with binary matrices marking statistically significant (*p* < 0.001) between the groups. **(b)** Average coherence matrices between EEG channel pairs for both groups and conditions, together with binary matrices highlighting channel pairs with significant intergroup differences (*p* < 0.001); red indicates pairs with a significant difference.

### 3.2 Subject-level classification results

The deep residual MLP model demonstrated moderate performance for mental fatigue detection using Leave-Subject-Out cross-validation, as summarized in Table 3. The results show substantial differences between the two experimental conditions:

**Table 3:** Subject-level binary classification metrics for deep residual MLP using LOSO-CV (*n* = 48).

Condition	Acc (%)	Bal Acc (%)	Prec (%)	Rec (%)	Spec (%)	F1 (%)	AUC (%)	CM
Music	72.92	65.00	70.73	96.67	33.33	81.69	69.63	(6, 12, 1, 29)
	[60–85]	–	–	–	–	[71–91]	[52–85]	
No Music	58.33	57.78	69.23	60.00	55.56	64.29	53.15	(10, 8, 12, 18)
	[46–73]	–	–	–	–	[49–78]	[36–71]	

Metrics include 95% bootstrap confidence intervals.

**Music condition:** The model achieved an accuracy of 72.92% across 48 subjects, with precision of 70.73% and notably high recall of 96.67%. The F1 score of 81.69% reflects a balance between precision and recall, though the model showed a tendency to over-predict the mental fatigue class. The AUC of 69.63% indicates modest discriminative ability in the presence of music stimulation.

**No-music condition:** Classification performance decreased substantially without music, achieving 58.33% accuracy with precision of 69.23% and recall of 60.00%. The F1 score of 64.29% and AUC of 53.15% suggest that the model struggled to effectively distinguish between mental fatigue states in the absence of musical stimulation. This performance is only marginally better than random chance, indicating that the no-music condition presents significant challenges for automated classification.

These results suggest several important observations. First, the presence of music during EEG recording appears to enhance the discriminability of mental fatigue states, potentially by modulating brain activity patterns in ways that make fatigue-related differences more pronounced, although the fixed condition order means order or habituation effects cannot be ruled out. Second, the high recall in the music condition (96.67%) indicates that the model successfully identifies most participants with mental fatigue, though at the cost of some false positives (reflected in lower precision). Third, the substantial performance gap between conditions highlights the importance of recording context in EEG-based mental health screening.

The subject-level aggregation approach (averaging predicted probabilities across all epochs per subject before thresholding) ensures that metrics reflect real-world clinical utility, where assessments are made at the individual level rather than on individual time windows. The LOSO cross-validation strategy provides realistic generalization estimates, as each subject is completely held out during training, mimicking deployment scenarios where the system encounters new individuals.

### 3.3 Feature importance and model interpretation

To understand which EEG features contribute most to mental fatigue classification, we applied SHAP (SHapley Additive exPlanations) analysis to the trained deep residual MLP model. SHAP values quantify the contribution of each feature to individual predictions, providing interpretable insights into the model's decision-making process.

In the music condition, the alpha-theta ratio features from frontal channels (Fp1, Fp2, and Fz) showed elevated SHAP importance, suggesting that the relationship between relaxation-associated alpha activity and drowsiness-associated theta activity is particularly informative when music is present. Additionally, theta-beta ratios from central electrodes (Cz) contributed significantly, potentially capturing arousal-related changes modulated by musical stimulation.

In the no-music condition, band power features from the alpha and beta bands showed relatively higher importance compared to the music condition, though overall feature discrimination was weaker (as reflected in the lower classification performance). This suggests that without the organizing influence of music on brain activity, the model relies more heavily on absolute power measures rather than relational features like ratios and coherence.

The combination of five complementary feature types (PSD, band powers, alpha-theta ratio, theta-beta ratio, and coherence) provided a comprehensive characterization of brain states, though the substantial performance difference between conditions indicates that the discriminability of these features depends critically on the recording context.

All experiments were conducted using Python 3.13 with PyTorch 2.8.0 (Python Software Foundation (PSF), Delaware, USA), scikit-learn 1.7.2, MNE 1.8, and the imbalanced-learn library. The random seed was fixed to 123 for all stochastic operations to ensure reproducibility. The concatenated feature vector comprised approximately 19,500 dimensions per epoch across all five feature types and 16 EEG channels. Model hyperparameters were selected through grid search with inner cross-validation on training folds, optimizing for validation AUC. The search space included learning rates [10^−5^, 10^−4^, and 10^−3^], batch sizes [8, 16, 32, 64, 128, and 256], dropout rates [0.1, 0.3, and 0.5], and hidden dimensions [512, 256, 128]. Training ran on a single NVIDIA GPU with approximately 2 mins per LOSO-CV fold.

### 3.4 Baseline model comparison

To contextualize the performance of the deep residual MLP, we compared it against three classical machine learning baselines: Random Forest (100 estimators), linear Support Vector Machine (SVM, LinearSVC with dual = False), and Logistic Regression (L2-regularized). All baseline models were evaluated using the same LOSO-CV protocol with SMOTE applied within each training fold. Given the high dimensionality of the concatenated feature set (approximately 19,500 features per epoch), PCA retaining 95% variance was applied prior to SVM and Logistic Regression to ensure computational tractability; Random Forest was run on the full feature set. Table 4 presents the results.

**Table 4:** Baseline model comparison for binary mental fatigue classification using LOSO-CV (*n* = 48).

Model	Condition	Acc (%)	Bal Acc (%)	Prec (%)	Rec (%)	AUC (%)
Deep Residual MLP	Music	**72.92**	65.00	70.73	**96.67**	**69.63**
Deep Residual MLP	No-music	58.33	57.78	69.23	60.00	53.15
Random Forest	Music	56.25	52.78	64.52	66.67	53.33
Random Forest	No-music	**70.83**	**65.56**	72.22	86.67	57.41
SVM (Linear, PCA)	Music	56.25	45.00	60.00	90.00	29.63
SVM (Linear, PCA)	No-music	45.83	36.67	55.00	73.33	21.30
Log. Reg. (PCA)	Music	60.42	50.56	62.79	90.00	40.56
Log. Reg. (PCA)	No-music	45.83	36.67	55.00	73.33	20.00

Deep Residual MLP results from Table 3 are included for reference. PCA (95% variance retained) was applied for SVM and Logistic Regression. Bold indicates best value per condition.

The baseline comparison reveals several important patterns. The deep residual MLP achieved the highest overall performance in the music condition (72.92% accuracy, 69.63% AUC), outperforming all classical baselines by a substantial margin. However, in the no-music condition, Random Forest unexpectedly outperformed the deep MLP (70.83% vs. 58.33% accuracy), suggesting that the non-linear ensemble approach better captured the weaker signal in the absence of music. The linear models (SVM and Logistic Regression) performed poorly across both conditions, with near-zero specificity (all participants classified as fatigued), indicating that the decision boundary between classes is highly non-linear. This pattern supports the use of deep architectures for EEG-based mental fatigue classification, particularly when music appeared to enhance neural discriminability.

### 3.5 Multi-output classification results

The multi-output classification extension of the deep residual MLP model simultaneously predicts anxiety, depression, and stress. While the binary classification task focused on overall mental fatigue detection, this extension provides insights into the individual psychological components that contribute to mental health assessment. Table 5 presents the multi-output classification performance metrics for the music condition.

**Table 5:** Multi-output classification performance using deep residual MLP with SMOTE in the music condition.

Component	Acc (%)	Bal Acc (%)	Prec (%)	Rec (%)	Spec (%)	F1 (%)	AUC (%)	CM
Anxiety	54.17	54.17	53.57	62.50	45.83	57.69	48.78	(11, 13, 9, 15)
Depression	62.50	55.56	50.00	27.78	83.33	35.71	58.70	(25, 5, 13, 5)
Stress	93.75	50.00	93.75	100.00	0.00	96.77	35.56	(0, 3, 0, 45)
Average	70.14	53.24	–	–	–	63.39	–	–

All metrics computed at the subject level using LOSO-CV (*n* = 48).

The results reveal substantial differences in classification performance across the three mental health components:

**Component-specific performance:** The model achieved moderate overall performance (average accuracy: 70.14%, average F1 score: 63.39%). Stress classification showed the highest accuracy (93.75%) with perfect recall (100.00%) and an F1 score of 96.77%; however, the AUC of 35.56% (below the chance level of 50%) indicates that the model's ranking ability for stress is poor. The high accuracy and F1 scores are attributable to the substantial class imbalance in the stress component (85.7% positive), as a model predicting the majority class for all subjects would achieve similarly high accuracy. The balanced accuracy for stress (50.00%, equal to random chance) confirms that the model's apparent performance on this component is entirely attributable to the 85.7% class prevalence. Depression classification achieved the second-highest accuracy (62.50%) but with notably low recall (27.78%), suggesting the model tends to under-predict depression cases. Anxiety showed moderate performance (54.17% accuracy, 57.69% F1 score) with reasonable recall (62.50%).

**Class imbalance considerations:** It is important to acknowledge that the dataset exhibits substantial class imbalance, particularly for stress classification where approximately 85.7% of subjects were classified as stressed. This imbalance likely contributed to the exceptionally high accuracy and F1 scores for stress detection, as the model can achieve high accuracy by predominantly predicting the majority class. The more balanced distributions for anxiety (49.0% positive) and depression (30.6% positive) resulted in more challenging classification tasks, as reflected in their lower performance metrics. The use of SMOTE (Synthetic Minority Over-sampling Technique) during training aimed to mitigate this imbalance, though evaluation was performed on the original unbalanced test sets to reflect real-world clinical distributions.

**SHAP-based feature importance analysis:** To understand which EEG features contribute most to each mental health component prediction, we applied SHAP (SHapley Additive exPlanations) analysis to the trained deep residual MLP model. Figure 4 presents the SHAP beeswarm plot summarizing feature contributions across the three output components. The plot reveals that anxiety predictions exhibit the widest spread of SHAP values, with some features showing substantial positive impact (up to 0.08), indicating that specific EEG patterns strongly influence anxiety classification. Depression shows notable outliers suggesting that certain features are highly informative for depression detection in specific individuals. Stress shows more concentrated SHAP distributions near zero, consistent with the high baseline prevalence in the dataset. The color gradient indicates feature values (red = high, blue = low), revealing that higher feature values generally correspond to positive SHAP contributions for anxiety and depression, while the relationship is more complex for stress.

**Figure 4:**
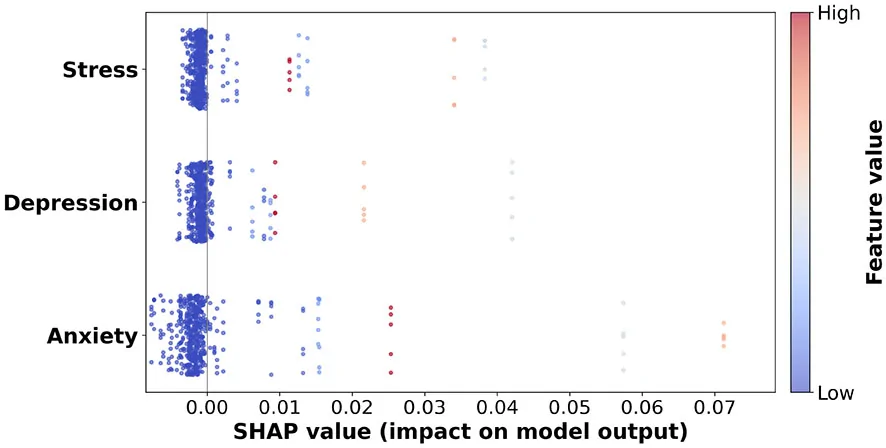
SHAP beeswarm plot showing feature importance and impact on model predictions for each mental health component (anxiety, depression, and stress) in the multi-output classification. The *x*-axis represents SHAP values (impact on model output), with positive values indicating features that push predictions toward the positive class. Color indicates feature value (red = high, blue = low). Anxiety shows the widest distribution of impactful features, while stress shows more concentrated contributions.

## 4 Discussion

The substantial performance difference between the music (72.92% accuracy) and no-music (58.33% accuracy) conditions is the central finding of this study, suggesting that music listening modulates brain activity in ways that make mental fatigue states more distinguishable. The no-music condition's near-chance performance (53.15% AUC) indicates that, without musical stimulation, the extracted EEG features do not capture sufficient discriminative information for reliable mental fatigue classification. This pattern is consistent with the notion that music engages attention, emotion, and memory networks (Koelsch, 2014; Menon and Levitin, 2005), thereby amplifying fatigue-related neural differences beyond what resting-state recordings reveal, and it underscores the importance of recording context for EEG-based mental health screening. However, because the condition order was fixed (no-music followed by music) for all participants, these differences may also reflect order, habituation, or time-related effects and cannot be attributed solely to music.

The high recall (96.67%) but lower precision (70.73%) in the music condition indicates that while the model successfully identifies most individuals with mental fatigue, it also produces false positives. This trade-off may be acceptable in screening applications where missing true cases is more costly than further evaluating false positives. However, for screening deployment, improved precision would be desirable.

It is important to acknowledge that our dataset exhibits class imbalance across the mental health components, particularly for stress, where approximately 85.7% of subjects were classified as stressed compared to more balanced distributions for anxiety (49.0% positive) and depression (30.6% positive). This imbalance produced the exceptionally high accuracy and F1 scores for stress detection (93.75% accuracy), which therefore reflect the prevalence of the stressed class rather than genuine discriminative ability; the more balanced distributions for anxiety and depression resulted in more challenging classification tasks. While SMOTE was applied during training to address this imbalance, future studies should aim for more balanced datasets through targeted recruitment strategies. Overall, the multi-output results indicate that the apparent success of the stress component is not evidence of a reliable stress-specific neural signature but rather an artifact of its high class prevalence.

This study demonstrated the application of a deep residual multi-layer perceptron (MLP), a feedforward deep learning architecture with residual skip connections, for EEG-based mental fatigue classification using Leave-Subject-Out cross-validation. Both binary classification (mental fatigue) and multi-output classification (anxiety, depression, and stress) were evaluated under music condition. The use of five complementary features (PSD, band powers, alpha-theta ratio, theta-beta ratio, and coherence) provided a comprehensive characterization of brain states, though their discriminative power varied substantially with target psychological component. The subject-level aggregation approach ensures that reported metrics reflect clinically meaningful performance at the individual level. The multi-output results demonstrate that EEG-based assessment can provide insights into specific psychological dimensions beyond the binary mental fatigue outcome, although the apparent success on the stress component reflects its high class prevalence rather than genuine discriminative ability, while anxiety and depression remain more challenging to classify. In conclusion, this preliminary study represents a step toward objective EEG-based mental health screening, though significant improvements in classification performance are needed before any clinical application. The findings underscore the importance of recording protocols and suggest that music-enhanced EEG may offer advantages for mental health assessment, although this interpretation requires confirmation in counterbalanced or randomized designs.

Future research should explore longitudinal studies with diverse populations, individual music preferences, and other physiological markers (e.g., heart rate variability) for a more comprehensive assessment. In addition, source-level analysis could provide deeper insights into the spatio-temporal frequency profiles of mental fatigue signatures in the brain. This could help identify the specific neural mechanism and networks and regions most affected by mental fatigue. Second, further investigation of non-brain artifacts that are typically removed during preprocessing could reveal additional markers of mental health status, as these signals might contain valuable physiological information related to mental state. Finally, validation of these findings across larger and more diverse populations will be crucial to developing robust and clinically applicable tools.

### 4.1 Limitations

Despite the moderate performance achieved in both binary and multi-output classification tasks, several limitations should be considered. First, the sample size (*n* = 48) is relatively small, and the study should be considered a preliminary investigation; larger cohorts are needed to validate these findings. Second, the condition order was fixed (no-music followed by music) for all participants, meaning that observed differences between conditions may reflect order effects, progressive fatigue, adaptation, or carry-over effects rather than music *per se*. Future studies should randomize or counterbalance condition order. Third, the dataset exhibits substantial class imbalance, particularly for stress classification (85.7% positive), which inflates accuracy and F1 scores for the majority class. Fourth, participants were recruited from a single university, limiting generalizability to other populations. Fifth, EEG recordings were short (5 mins per condition) and used a single music track, which may not capture the full range of music-induced neural modulation. Sixth, labels were assigned based on a standardized fatigue questionnaire and a predefined cutoff rather than formal psychiatric diagnosis, and the study does not constitute a clinical diagnostic tool. Seventh, dry electrodes were used, which have higher impedance and are more susceptible to artifacts than wet electrodes, particularly in the gamma band. Finally, no external or independent validation dataset was available to confirm model performance.

## Data Availability

The raw data supporting the conclusions of this article will be made available by the authors, without undue reservation.
