# Supplementary material for: Music-evoked EEG signals for mental health assessment using two- and three-class classification
Source: Front Syst Neurosci. 2026 Sep 23;20:1886805. doi: 10.3389/fnsys.2026.1886805 (PMC13642751; doi:10.3389/fnsys.2026.1886805)
Supplement: Supplementary file 1 [file Data_Sheet_1.pdf]

Supplementary Material

1 SUPPLEMENTARY FIGURES

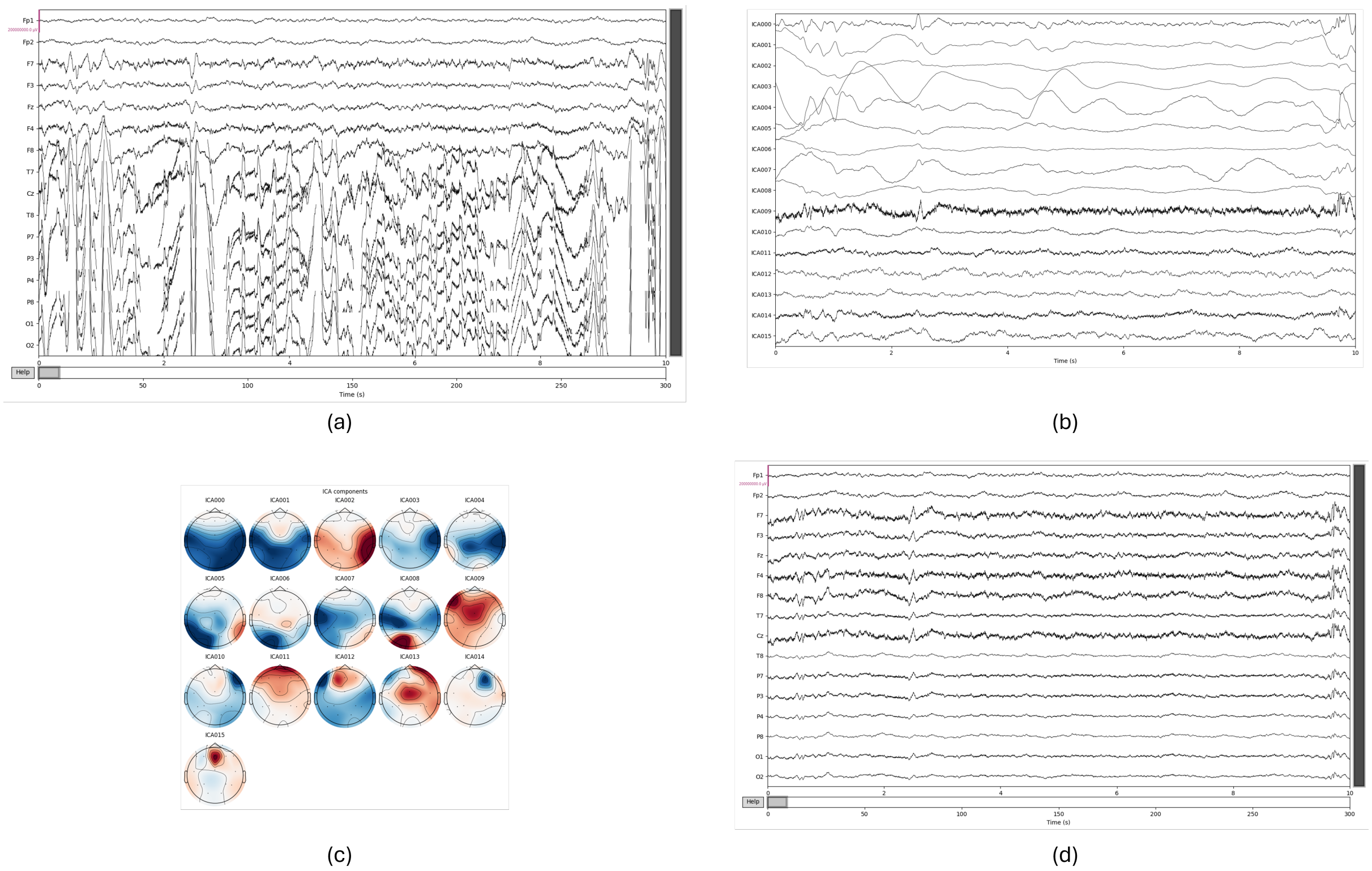

Figure S1. Plots of the preprocessing pipeline for a representative Subject 1. (a) Filtered EEG signal, (b) Independent components extracted using ICA, (c) Heatmaps of the extracted ICs, and (d) Reconstructed EEG signal.

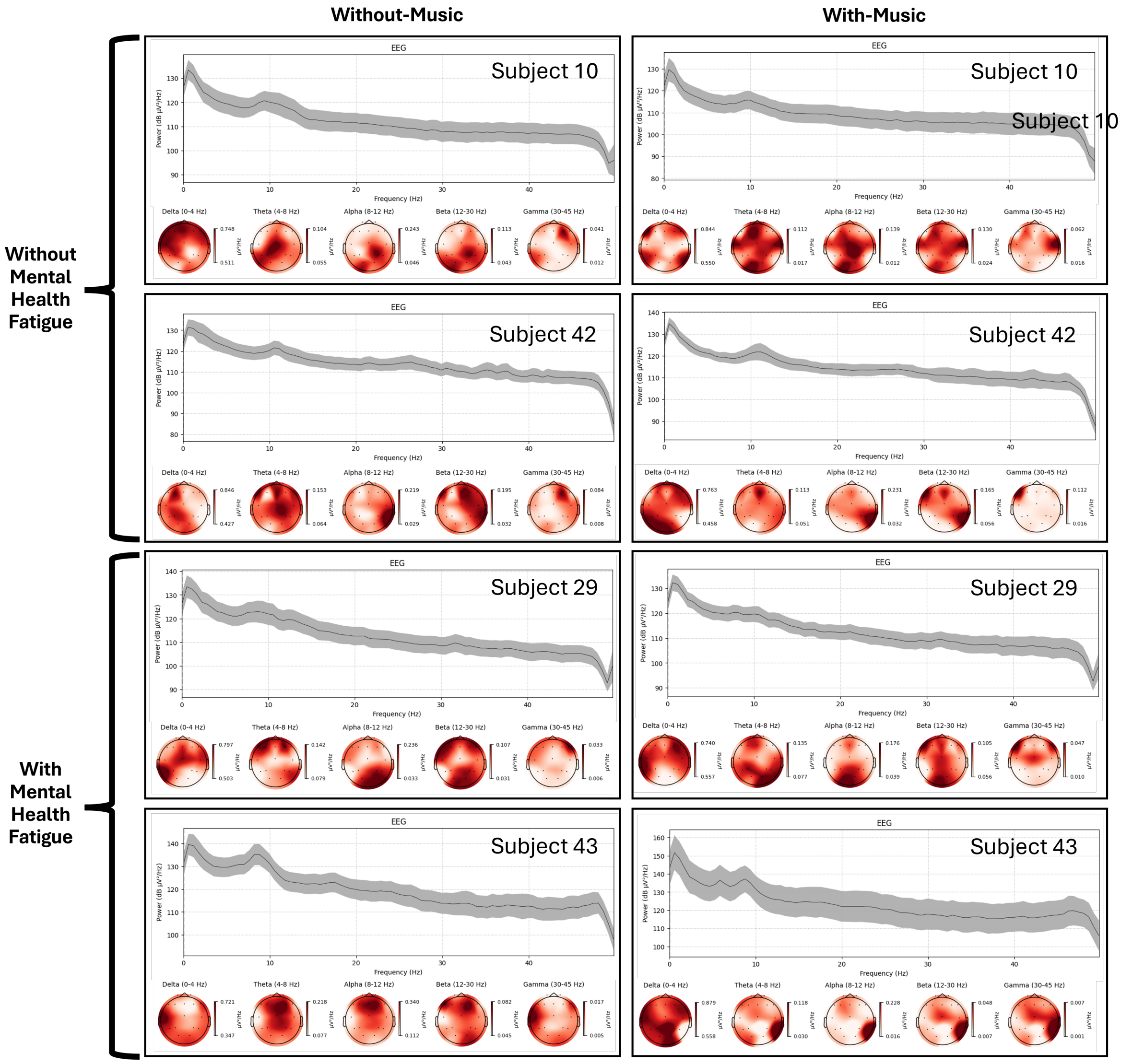

Figure S2. EEG spectral power distribution and topographic maps across frequency bands (delta, theta, alpha, beta, and gamma) for representative participants ($n = 4$) with and without mental health fatigue, shown in both music and no-music conditions. The line plots show power spectral density (PSD) with confidence intervals, while the topographic maps illustrate the spatial distribution of power for each frequency band. Darker red indicates higher power values. The selected participants demonstrate typical patterns observed in the study.

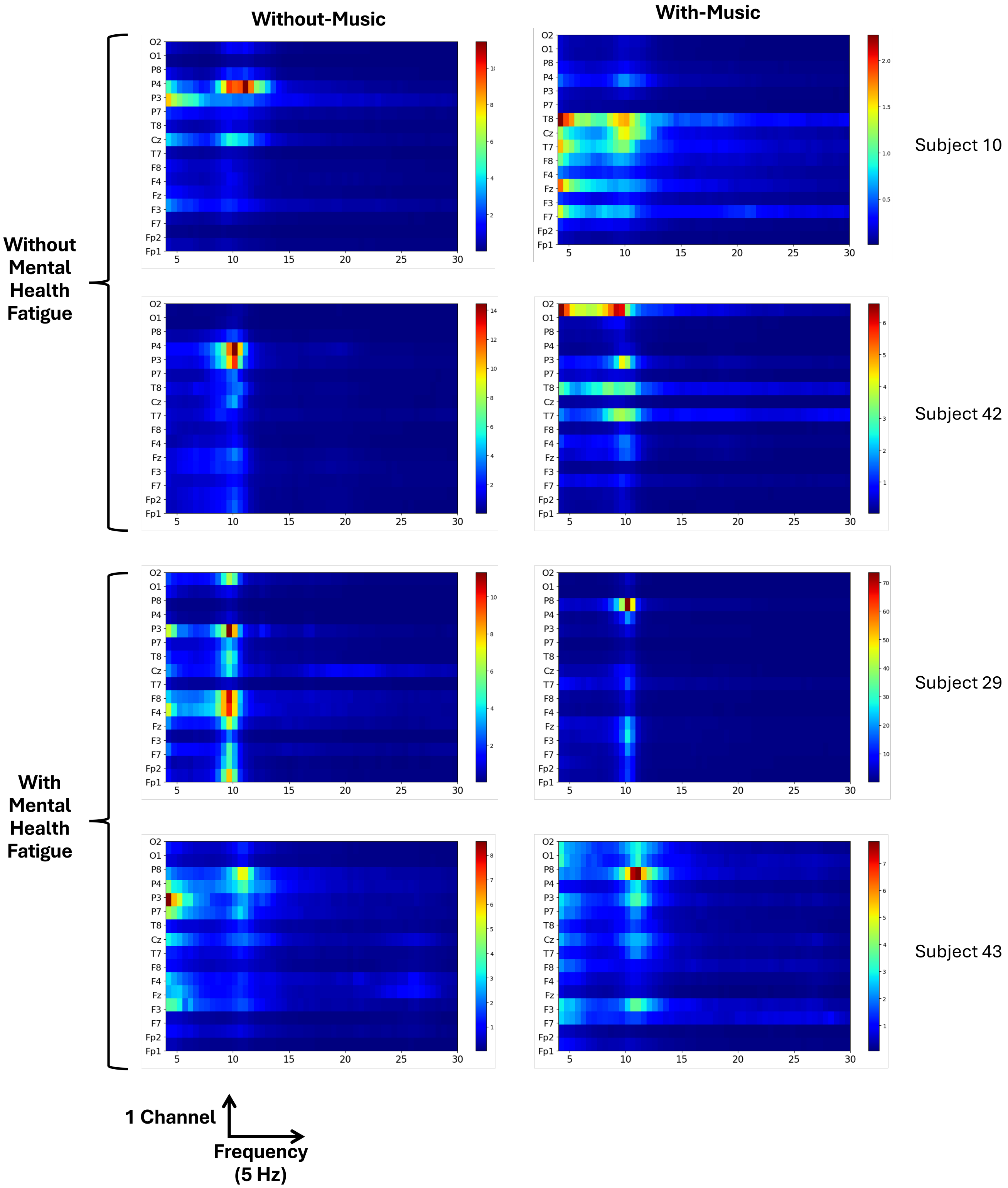

Figure S3. Power Spectral Density (PSD) averages of EEG data for four selected subjects under different states. The PSD averages highlight representative variations in neural activity across frequency bands influenced by music and mental health fatigue in the selected subjects.

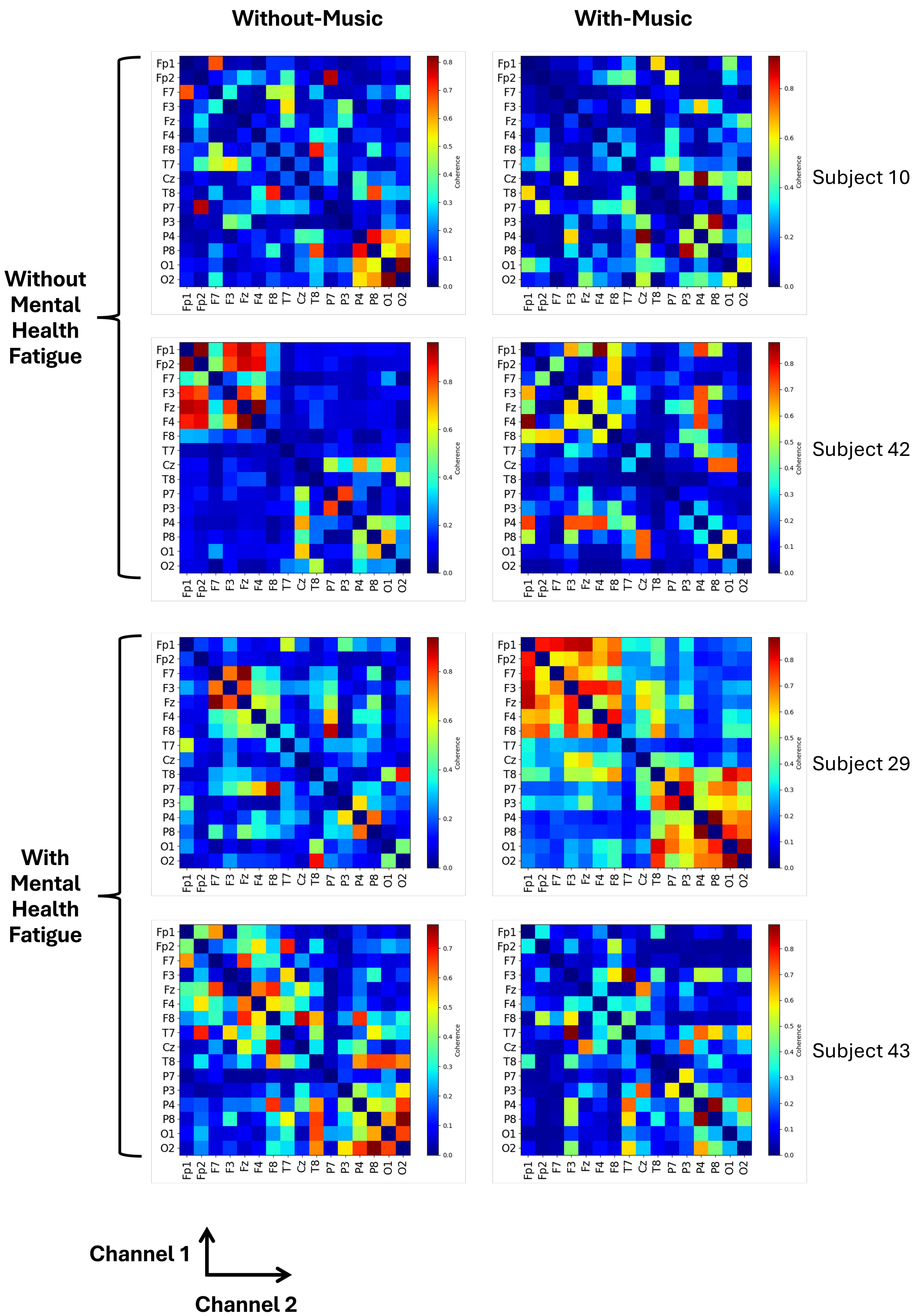

Figure S4. Coherence matrices of EEG data for four selected subjects under different states. The matrices illustrate connectivity patterns between EEG channels, showcasing representative variations influenced by music and mental health fatigue across the selected subjects.

2 APPENDIX A: SURVEY QUESTIONS

2.1 Generalized Anxiety Disorder 7-item (GAD-7) Scale

Over the last 2 weeks, how often have you been bothered by the following problems?

Response options: 0 = Not at all, 1 = Several days, 2 = More than half the days, 3 = Nearly every day

1. Feeling nervous, anxious, or on edge
2. Not being able to stop or control worrying
3. Worrying too much about different things
4. Trouble relaxing
5. Being so restless that it's hard to sit still
6. Becoming easily annoyed or irritable
7. Feeling afraid as if something awful might happen

2.2 Patient Health Questionnaire-9 (PHQ-9)

Over the last 2 weeks, how often have you been bothered by any of the following problems?

Response options: 0 = Not at all, 1 = Several days, 2 = More than half the days, 3 = Nearly every day

1. Little interest or pleasure in doing things
2. Feeling down, depressed, or hopeless
3. Trouble falling or staying asleep, or sleeping too much
4. Feeling tired or having little energy
5. Poor appetite or overeating
6. Feeling bad about yourself — or that you are a failure or have let yourself or your family down
7. Trouble concentrating on things, such as reading the newspaper or watching television
8. Moving or speaking so slowly that other people could have noticed. Or the opposite — being so fidgety or restless that you have been moving around a lot more than usual
9. Thoughts that you would be better off dead, or of hurting yourself

2.3 Multidimensional Fatigue Scale (MFS)

The following questions ask about your level of fatigue. For each question, please indicate how you have been feeling during the past week.

Response options: 1 = Not at all, 2 = A little, 3 = Moderately, 4 = Quite a bit, 5 = Extremely

1. I feel fatigued
2. I feel weak all over
3. I feel listless
4. I feel tired
5. I have trouble starting things because I am tired
6. I have trouble finishing things because I am tired
7. I have energy

8. I am able to do my usual activities
9. I need to sleep during the day
10. I am too tired to eat
11. I need help doing my usual activities
12. I am frustrated by being too tired to do the things I want to do
13. I have to limit my social activity because I am tired

2.4 Perceived Stress Scale (PSS)

The questions in this scale ask you about your feelings and thoughts during the last month. In each case, you will be asked to indicate how often you felt or thought a certain way.

Response options: 0 = Never, 1 = Almost Never, 2 = Sometimes, 3 = Fairly Often, 4 = Very Often

1. In the last month, how often have you been upset because of something that happened unexpectedly?
2. In the last month, how often have you felt that you were unable to control the important things in your life?
3. In the last month, how often have you felt nervous and stressed?
4. In the last month, how often have you felt confident about your ability to handle your personal problems?
5. In the last month, how often have you felt that things were going your way?
6. In the last month, how often have you found that you could not cope with all the things that you had to do?
7. In the last month, how often have you been able to control irritations in your life?
8. In the last month, how often have you felt that you were on top of things?
9. In the last month, how often have you been angered because of things that happened that were outside of your control?
10. In the last month, how often have you felt difficulties were piling up so high that you could not overcome them?
